# Hydrogel-Based Delivery Systems for Non-Opioid Analgesics: Advances, Challenges, and Clinical Prospects

**DOI:** 10.3390/jcm14217768

**Published:** 2025-11-01

**Authors:** Kyung Kwan Lee, Wonwoo Jeong, Minsuk Chae

**Affiliations:** 1 Department of Bio-Chemical Engineering, Chosun University, 309 Pilmun-daero, Dong-gu, Gwangju 61452, Republic of Korea; kylee@chosun.ac.kr; 2 Wake Forest Institute for Regenerative Medicine, Wake Forest School of Medicine, Winston-Salem, NC 27157, USA; wjeong@wakehealth.edu; 3 Department of Anesthesiology and Pain Medicine, College of Medicine, The Catholic University of Korea, Seoul 06591, Republic of Korea

**Keywords:** hydrogel, non-opioid analgesics, stimulus-responsive, sustained release, pain management, drug delivery, biocompatibility

## Abstract

This review focuses on hydrogel-based systems specifically designed for non-opioid analgesics, aiming to improve efficacy, safety, and translational applicability. The opioid crisis has intensified the need for safer and more effective alternatives in pain management. Non-opioid analgesics including NSAIDs, acetaminophen, gabapentinoids, antidepressants, anticonvulsants, NMDA receptor antagonists, topical agents, and cannabinoids offer promising options but are limited by rapid clearance, short half-lives, and off-target effects. Hydrogel-based drug delivery systems present a novel solution by enabling controlled, localized, and sustained release of analgesics, thus improving therapeutic efficacy and minimizing systemic toxicity. Advances in stimulus-responsive, self-healing, mechanically robust, and hybrid or nanocomposite hydrogels have broadened their biomedical applications and clinical relevance. This narrative review summarizes key hydrogel technologies and their integration with non-opioid analgesic agents. We explore encapsulation strategies, drug release mechanisms, and emerging clinical data, while also addressing critical challenges such as biocompatibility, mechanical durability, and translational scalability. Interdisciplinary collaboration between material scientists, clinicians, and regulatory experts is essential to advance hydrogel-based therapies from bench to bedside. Overall, hydrogel platforms hold transformative potential in optimizing non-opioid analgesic delivery and redefining the future of pain management.

## 1. Introduction

Pain management remains a significant global healthcare challenge, impacting patient morbidity, healthcare utilization, and quality of life. Opioid analgesics have traditionally served as the primary treatment for moderate to severe pain; however, their use is associated with serious adverse effects, including tolerance, dependence, addiction, respiratory depression, gastrointestinal issues, and overdose risk [1,2,3]. The escalating opioid epidemic underscores the urgent need for safer and more effective alternatives. Non-opioid analgesics including nonsteroidal anti-inflammatory drugs (NSAIDs), acetaminophen, gabapentinoids, antidepressants, anticonvulsants, NMDA receptor antagonists, topical agents, and cannabinoids have gained increasing attention due to their improved safety profiles and lower risk of abuse. Nevertheless, their therapeutic potential is often limited by rapid systemic clearance, short half-lives, low bioavailability, and non-specific distribution, which can lead to suboptimal efficacy and systemic adverse effects [4,5,6,7,8]. To address these pharmacokinetic and pharmacodynamic limitations, innovative drug delivery technologies are required to provide sustained, localized, and controlled release of analgesics [9].

Hydrogels are three-dimensional, cross-linked polymer networks capable of absorbing large amounts of water or biological fluids. They have emerged as promising biomaterials for advanced drug delivery due to their inherent biocompatibility, biodegradability, structural similarity to the extracellular matrix (ECM), and tunable physicochemical properties. These characteristics enable efficient encapsulation and sustained release of various bioactive agents, supporting localized delivery, enhanced efficacy, reduced dosing frequency, and fewer systemic side effects. Recent advances in polymer chemistry and biomedical engineering have driven significant progress in hydrogel-based drug delivery systems [9,10,11]. Innovations include stimulus-responsive hydrogels that release drugs in response to environmental cues such as pH, temperature, enzymatic activity, or ionic changes [12]; self-healing hydrogels that restore structural integrity under physiological stress [13]; and mechanically robust hydrogels designed to function in dynamic or load-bearing biological environments [14]. Hybrid hydrogels, which combine natural and synthetic polymers, further enhance biocompatibility, mechanical strength, and controlled release capabilities [15].

Integrating advanced hydrogel technologies with non-opioid analgesics offers significant potential to overcome current limitations in drug delivery. Recent studies support this approach, demonstrating improved therapeutic outcomes. NSAID-loaded hydrogels have shown enhanced analgesic and anti-inflammatory effects, along with reduced gastrointestinal side effects compared to conventional formulations [16]. Gabapentinoid-containing hydrogels have achieved sustained, targeted analgesia in neuropathic pain models while minimizing systemic adverse effects such as sedation [17,18]. Similarly, hydrogels delivering antidepressants or ketamine have prolonged analgesic duration and improved patient tolerance through reduced systemic exposure. Moreover, nanocomposite hydrogels incorporating nanoparticles, liposomes, or micelles and biofunctionalized hydrogels containing cell-adhesion motifs or growth factors, exhibit multifunctional properties. These systems can co-deliver analgesics with adjunct therapeutic agents, enhancing their clinical utility in complex settings such as chronic pain and tissue regeneration [19].

Despite notable progress, several critical challenges hinder the clinical translation of hydrogel-based non-opioid analgesic delivery systems. These include optimizing drug encapsulation efficiency, achieving precise control over release kinetics, improving biocompatibility, enhancing mechanical stability, and navigating complex regulatory pathways [9,10,11]. Overcoming these barriers requires a multidisciplinary approach that integrates expertise from clinicians, pharmacologists, biomedical engineers, and regulatory professionals.

**Figure 1:**
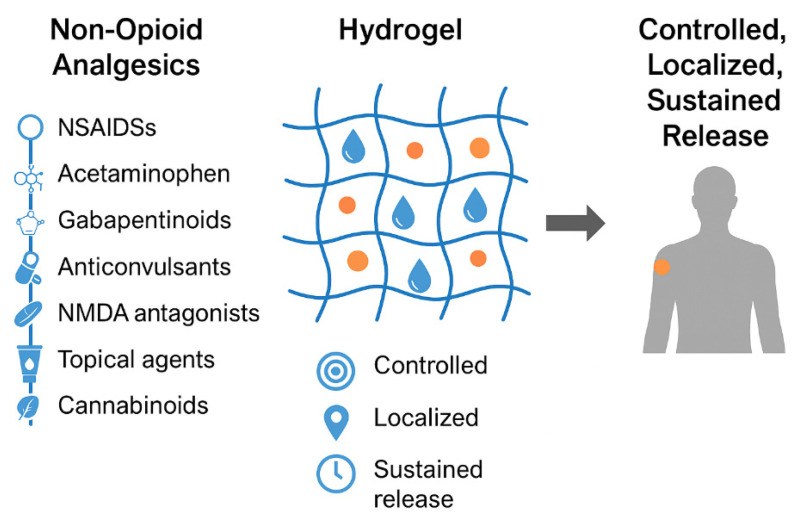
Schematic illustration of hydrogel-based delivery systems for non-opioid analgesics.

This narrative review explores recent advances in hydrogel-based drug delivery systems designed for non-opioid analgesics (Figure 1). It examines key aspects including physicochemical properties, encapsulation methods, and controlled release mechanisms, while evaluating relevant preclinical and clinical findings. In addition, the review addresses major translational challenges, regulatory considerations, and outlines future research directions critical for clinical implementation. By emphasizing interdisciplinary collaboration, it underscores the transformative potential of hydrogel technologies in enhancing non-opioid analgesic therapy and improving pain management outcomes.

## 2. Non-Opioid Analgesic Drugs

Non-opioid analgesics comprise a diverse group of agents used increasingly to manage pain without the adverse effects commonly associated with opioids. Their growing clinical use is driven by favorable safety profiles, reduced risk of dependence, and proven efficacy in treating both acute postoperative pain and chronic neuropathic or inflammatory conditions. However, their effectiveness can be limited by short half-lives, rapid systemic clearance, and insufficient localization at target sites [4,5,6]. Advances in pharmaceutical science and drug delivery technologies particularly hydrogel-based systems have helped address these limitations by enhancing drug retention, prolonging analgesic effects, and minimizing systemic side effects [20]. Despite their pharmacological efficacy, most non-opioid analgesics suffer from poor bioavailability, short half-lives, and insufficient localization at target sites. Hydrogel-based delivery systems offer a promising solution by enabling localized, sustained, and biocompatible release of these drugs. Therefore, integrating hydrogel technology into non-opioid analgesic delivery represents a rational and translational approach to improving therapeutic outcomes and minimizing systemic toxicity. This section reviews the major classes of non-opioid analgesics, including NSAIDs, acetaminophen, gabapentinoids, antidepressants, anticonvulsants, NMDA receptor antagonists, topical agents, and cannabinoids, with a focus on their mechanisms, clinical applications, and recent innovations in formulation and delivery technologies (Table 1) [8,21].

### 2.1. Nonsteroidal Anti-Inflammatory Drugs (NSAIDs)

NSAIDs, including ibuprofen, naproxen, diclofenac, and selective COX-2 inhibitors like celecoxib, are commonly used to treat acute and chronic inflammatory conditions due to their analgesic and anti-inflammatory effects. Their primary mechanism involves inhibition of cyclooxygenase (COX) enzymes, preventing the conversion of arachidonic acid into prostaglandins key mediators of inflammation, pain, and fever [22]. Non-selective NSAIDs inhibit both COX-1 and COX-2, often resulting in gastrointestinal side effects due to COX-1 inhibition, while COX-2 selective inhibitors reduce this risk and are preferred for patients with gastrointestinal vulnerability [22,23]. Clinically, NSAIDs are widely used for osteoarthritis, rheumatoid arthritis, postoperative pain, dysmenorrhea, and musculoskeletal injuries. Recent strategies combining NSAIDs with gastroprotective agents have further improved safety profiles [23]. In parallel, hydrogel-based delivery systems have emerged as innovative platforms to enhance NSAID therapy. Preclinical studies show that NSAID-loaded hydrogels provide prolonged analgesia, improved drug localization, reduced dosing frequency, and minimized systemic exposure, leading to better patient compliance and safety compared to conventional formulations [16].

### 2.2. Acetaminophen (Paracetamol)

Acetaminophen, or paracetamol, is widely used as a centrally acting analgesic and antipyretic. Its primary mechanism involves inhibition of central prostaglandin synthesis, possibly through COX enzyme modulation in the central nervous system [24]. Other proposed mechanisms include interaction with serotonergic and endocannabinoid pathways. Clinically, acetaminophen is preferred for mild to moderate pain and fever, especially when NSAIDs are contraindicated due to gastrointestinal risks. Its favorable safety profile has made it a core component of multimodal analgesia, particularly in perioperative settings [25]. Recent developments include intravenous formulations for rapid analgesia and reduced opioid use. Additionally, hydrogel-based acetaminophen systems have been designed to enable sustained, localized drug release. These formulations enhance analgesic efficacy, extend duration of action, and lower hepatotoxicity risk by minimizing systemic exposure [26,27], thereby improving overall safety, compliance, and clinical outcomes.

### 2.3. Gabapentinoids

Gabapentinoids, including gabapentin and pregabalin, provide analgesia by binding to the alpha-2-delta subunit of voltage-gated calcium channels in the central nervous system. This reduces calcium influx and inhibits the release of excitatory neurotransmitters such as glutamate and substance P [28,29]. Clinically, gabapentinoids are effective in treating neuropathic pain conditions, including diabetic peripheral neuropathy, postherpetic neuralgia, spinal cord injury-associated pain, and fibromyalgia. They are also increasingly used in multimodal perioperative analgesia to reduce opioid use and improve postoperative outcomes [30,31]. Hydrogel-based gabapentinoid delivery systems have demonstrated promising results in preclinical and clinical studies, offering sustained local release, enhanced bioavailability, and fewer systemic side effects such as sedation and dizziness. These formulations improve treatment adherence, patient comfort, and overall outcomes in neuropathic pain management [32,33,34].

### 2.4. Antidepressants

Analgesic antidepressants include tricyclic antidepressants (TCAs) such as amitriptyline and nortriptyline, and serotonin-norepinephrine reuptake inhibitors (SNRIs) like duloxetine and venlafaxine. These agents primarily modulate central serotonin and norepinephrine pathways to enhance descending inhibitory pain signals. TCAs also block sodium and calcium channels, contributing additional analgesic effects [35]. Clinically, antidepressants are used to treat various chronic neuropathic pain conditions, including diabetic neuropathy, postherpetic neuralgia, fibromyalgia, and complex regional pain syndrome. They are particularly beneficial for patients with comorbid mood disorders, addressing both pain and emotional symptoms. Their use has also expanded to chemotherapy-induced neuropathic pain, with studies showing reduced pain severity and improved quality of life [36,37]. Advances in drug delivery, especially hydrogel-based systems, have further enhanced the utility of antidepressants by enabling sustained, localized release. These formulations reduce systemic side effects such as sedation, dry mouth, and cardiovascular issues, while improving adherence and therapeutic outcomes in neuropathic pain management [38,39].

### 2.5. Anticonvulsants

Anticonvulsants such as carbamazepine, lamotrigine, topiramate, oxcarbazepine, and valproic acid possess notable analgesic properties. Their mechanisms include inhibition of voltage-gated sodium channels (e.g., carbamazepine, lamotrigine, oxcarbazepine), modulation of glutamate release, and enhancement of GABA-mediated inhibitory transmission (e.g., topiramate, valproic acid) [40]. Clinically, these agents are effective in treating neuropathic pain syndromes such as trigeminal neuralgia, diabetic neuropathy, and central pain due to spinal cord injury or stroke. They are also used for migraine prophylaxis and fibromyalgia management, reflecting broad therapeutic utility [41,42]. Recent advances in hydrogel-based delivery systems have improved anticonvulsant therapy by enabling sustained, site-specific drug release. Gabapentinoids such as gabapentin and pregabalin are structurally derived from anticonvulsants but have distinct analgesic indications, particularly in neuropathic pain. These formulations enhance efficacy, reduce systemic side effects, and support better adherence and quality of life by maintaining consistent therapeutic levels at the target site [43,44].

### 2.6. NMDA Receptor Antagonists

NMDA receptor antagonists, such as ketamine, exert analgesic effects by inhibiting the N-methyl-D-aspartate (NMDA) receptor, which plays a key role in pain perception, transmission, and central sensitization. Clinically, low-dose ketamine is increasingly used for acute postoperative pain, chronic neuropathic pain, and opioid-resistant conditions due to its strong analgesic effect and opioid-sparing potential [21,45]. Its therapeutic applications have expanded to treatment-resistant depression and complex regional pain syndrome (CRPS), showing significant improvements in both pain relief and quality of life [46,47]. Recent preclinical studies on hydrogel-based ketamine delivery systems demonstrate their ability to provide sustained, targeted release, enhancing analgesic efficacy while reducing systemic psychotomimetic side effects. These formulations improve patient adherence and overall treatment outcomes [48,49].

### 2.7. Topical Agents

Topical analgesics such as capsaicin and lidocaine patches provide localized pain relief by directly targeting peripheral nociceptors. Capsaicin reduces pain by depleting substance P from sensory nerve endings, making it effective for conditions like postherpetic neuralgia and peripheral neuropathic pain [50,51]. Lidocaine acts by blocking local voltage-gated sodium channels, attenuating neuropathic pain signals, and is commonly used for diabetic neuropathy and localized nerve-related pain [52]. High-concentration capsaicin patches have been developed to extend analgesic duration and reduce application frequency, thereby improving patient compliance [53]. Hydrogel-based topical formulations further enhance drug penetration, prolong therapeutic effects, and minimize systemic absorption. These advanced systems offer improved efficacy, greater patient comfort, and wider clinical applicability compared to conventional topical agents [54].

### 2.8. Cannabinoids

Cannabinoids such as tetrahydrocannabinol (THC) and cannabidiol (CBD) exert analgesic effects by interacting with the endocannabinoid system, primarily through CB1 and CB2 receptors. Clinically, they are increasingly used to manage chronic pain conditions including neuropathic pain, multiple sclerosis-related spasticity, and refractory pain unresponsive to standard therapies [55,56]. Recent studies highlight their analgesic potential and associated improvements in patient quality of life. Ongoing research focuses on optimizing dosing and formulations to enhance efficacy and reduce adverse effects, particularly the cognitive and psychomimetic effects linked to THC [57,58]. Hydrogel-based delivery systems offer a promising approach by enabling sustained, localized cannabinoid release while minimizing systemic exposure [59]. These formulations improve therapeutic precision, enhance patient adherence, and address limitations of conventional cannabinoid delivery methods [60,61].

Collectively, these non-opioid analgesic classes provide valuable alternatives to opioids but still face pharmacological and clinical limitations such as short half-lives, systemic side effects, and inconsistent efficacy in chronic pain. These shortcomings highlight the unmet need for advanced formulation technologies such as hydrogels that can improve drug localization, prolong analgesic effects, and reduce systemic exposure.

## 3. Fundamentals and Advanced Concepts in Hydrogel Technologies

### 3.1. Definition, Basic Principles, and Characteristics of Hydrogels

#### 3.1.1. Definition

Hydrogels are hydrophilic, three-dimensional polymer networks capable of absorbing and retaining large amounts of water or biological fluids while preserving their structural integrity. Their softness, elasticity, and high-water content allow them to closely mimic the natural extracellular matrix (ECM), supporting biocompatibility and favorable cellular interactions. These biomimetic properties make hydrogels ideal candidates for various biomedical applications, including drug delivery, tissue engineering, wound healing, and regenerative medicine [62].

#### 3.1.2. Basic Principles

Hydrogels consist of polymer chains interconnected through chemical or physical cross-linking mechanisms. Chemically cross-linked hydrogels form stable, covalent-bonded networks, whereas physically cross-linked hydrogels rely on transient interactions such as ionic bonds, hydrogen bonds, or hydrophobic associations. These cross-linking strategies significantly influence the mechanical strength, swelling behavior, and degradation profiles of hydrogels [63]. Their intrinsic hydrophilicity arises from abundant hydrophilic groups (e.g., hydroxyl, carboxyl, amide, and sulfate), enabling substantial water absorption. Factors such as polymer concentration, molecular weight, and cross-linking density regulate swelling and hydration kinetics. Additionally, hydrogels exhibit viscoelastic properties, demonstrating combined elastic and viscous responses under mechanical stress. This viscoelastic behavior closely mimics biological tissues and supports essential biological functions, including cellular adhesion, proliferation, and differentiation, crucial for biomedical applications [64,65].

### 3.2. Key Characteristics

#### 3.2.1. Physicochemical Properties

Hydrogels exhibit several distinctive physicochemical properties that underpin their diverse biomedical applications. Their highly porous structure facilitates efficient nutrient, oxygen, and metabolite transport, promoting cellular infiltration and tissue integration essential for regenerative medicine and tissue engineering. Porosity, pore size, and interconnectivity can be precisely tuned via polymer selection and fabrication techniques [66,67]. Additionally, hydrogels offer adjustable mechanical characteristics including stiffness, elasticity, toughness, and resilience by varying polymer types, molecular weights, cross-linking density, and synthesis conditions. This mechanical versatility enables hydrogels to match properties of diverse biological tissues, ranging from soft tissues (e.g., skin, cartilage) to load-bearing structures (e.g., bone) [68,69]. Biocompatibility and biodegradability are also critical attributes. Natural polymer-based hydrogels (e.g., alginate, chitosan, gelatin, collagen, hyaluronic acid) inherently possess superior biocompatibility due to their ECM-mimetic nature. Conversely, synthetic hydrogels (e.g., polyethylene glycol [PEG], polyvinyl alcohol [PVA], poly(lactic-co-glycolic acid) [PLGA]) offer precise mechanical and degradation control but often require biofunctionalization or surface modification to enhance biocompatibility [70,71]. Hydrogels additionally provide selective permeability and controlled diffusion characteristics, essential for effective encapsulation and sustained release of therapeutic agents. Advanced surface functionalization methods, such as immobilizing bioactive molecules (peptides, growth factors), further enhance their bioactivity and therapeutic performance [72,73,74].

#### 3.2.2. Stimulus-Responsive Behaviors (Smart Hydrogels)

**Figure 2:**
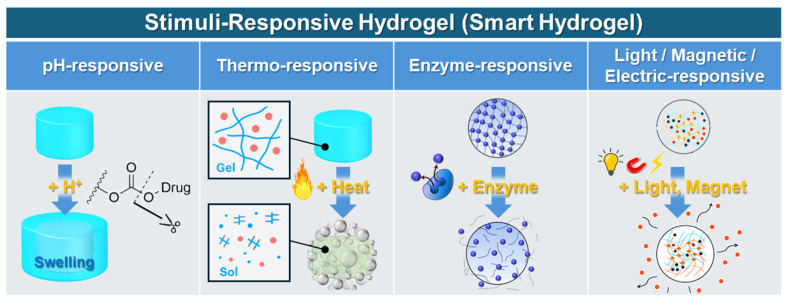
Stimulus-responsive hydrogels and their drug release mechanisms.

Stimulus-responsive or “smart” hydrogels represent significant advancements in hydrogel technology, exhibiting dynamic physicochemical changes in response to specific environmental or biological stimuli. These materials provide precise spatial and temporal control over drug release and hydrogel properties, enhancing therapeutic efficacy and minimizing systemic side effects [12,73,75]. pH-responsive hydrogels contain ionizable groups that reversibly respond to pH fluctuations through protonation/deprotonation, making them ideal for targeted drug delivery in pathological conditions with altered pH, such as tumors, inflammatory regions, or specific gastrointestinal compartments [12,67,76]. Thermo-responsive hydrogels exhibit sol–gel transitions triggered by temperature shifts based on lower critical solution temperature (LCST) or upper critical solution temperature (UCST). Common examples, such as poly(N-isopropylacrylamide) (PNIPAAm) and Pluronic^®^ hydrogels, form injectable solutions that solidify at physiological temperatures, enabling minimally invasive applications [77,78]. Ion-responsive hydrogels utilize ionic interactions and cross-linking by divalent cations (Ca^2+^, Mg^2+^), allowing controlled swelling and release behaviors tailored to specific ionic environments. This responsiveness is advantageous, particularly with natural polymer hydrogels such as alginate [79,80]. Enzyme-responsive hydrogels incorporate peptide sequences that selectively degrade upon exposure to enzymes (e.g., matrix metalloproteinases) overexpressed in certain pathological states, ensuring targeted and controlled therapeutic release [81,82]. Light-responsive hydrogels integrate photolabile groups or photo-crosslinkable components, enabling controlled modulation of hydrogel properties and drug release by external irradiation (UV, visible, near-infrared). This method provides remote, non-invasive control of therapeutic outcomes [83]. Redox-responsive hydrogels contain oxidation-sensitive linkages (e.g., disulfide bonds) that cleave under intracellular glutathione conditions, making them valuable for targeted intracellular drug delivery in cancerous or diseased cells [84]. Finally, magnetic and electric field-responsive hydrogels incorporate magnetic nanoparticles or conductive polymers, respectively, allowing remote and reversible manipulation of hydrogel deformation, drug release kinetics, and mechanical characteristics through external magnetic or electrical fields. This capability expands their potential into advanced biomedical applications requiring precise, remotely controlled therapeutic delivery (Figure 2) [85].

### 3.3. Types of Hydrogels

Hydrogels can be categorized into three primary groups based on their polymer origin and compositional properties: natural, synthetic, and hybrid hydrogels. Each category demonstrates unique physicochemical characteristics, biological interactions, and clinical potential, providing distinct advantages and limitations for specific biomedical applications (Table 2) [63].

#### 3.3.1. Natural Hydrogels

Natural hydrogels are derived from biological biopolymers, including polysaccharides (e.g., alginate, chitosan, hyaluronic acid, agarose), proteins (e.g., gelatin, collagen, fibrin, silk fibroin), and glycosaminoglycans. Their inherent biocompatibility, biodegradability, and similarity to native extracellular matrix (ECM) components make them highly suitable for biomedical applications such as drug delivery, tissue engineering, wound healing, and regenerative medicine [86,87,88]. For instance, alginate hydrogels rapidly gelate upon exposure to divalent cations (Ca^2+^, Mg^2+^), enabling gentle encapsulation of drugs or cells, making them ideal for injectable systems, wound dressings, and tissue regeneration scaffolds. Hyaluronic acid-based hydrogels closely resemble ECM, supporting cellular adhesion, proliferation, and differentiation, particularly in cartilage regeneration and dermal tissue engineering [89]. Despite these advantages, natural hydrogels have limitations, including batch-to-batch variability, relatively weak mechanical strength, rapid biodegradation, and potential immunogenicity or pathogen contamination. These limitations necessitate meticulous purification and standardization during production [90,91].

#### 3.3.2. Synthetic Hydrogels

Synthetic hydrogels are chemically engineered from polymers such as polyethylene glycol (PEG), polyvinyl alcohol (PVA), poly(lactic-co-glycolic acid) (PLGA), polyacrylamide (PAAm), poly(acrylic acid) (PAA), and poly(N-isopropylacrylamide) (PNIPAAm). They offer distinct advantages over natural hydrogels, including reproducible synthesis, precise control over mechanical properties, adjustable degradation rates, and minimal risk of biological contamination. Their physicochemical properties such as stiffness, porosity, and swelling can be precisely tailored through polymerization and cross-linking techniques [92]. PEG-based hydrogels are widely used in drug delivery and tissue engineering due to their highly customizable cross-linking density, excellent biocompatibility, and minimal immunogenicity. PNIPAAm hydrogels exhibit thermo-responsive behavior, undergoing phase transitions around physiological temperatures, enabling injectable, in situ-forming gels for minimally invasive therapeutic delivery [93]. However, synthetic hydrogels typically lack inherent bioactivity, cell-adhesion capabilities, and biofunctional cues necessary for effective biological integration. To overcome these limitations, bioactive molecules, peptides (e.g., RGD peptides), or growth factors are frequently conjugated or immobilized onto hydrogel surfaces, significantly enhancing their biological interactions and therapeutic efficacy [94].

#### 3.3.3. Hybrid Hydrogels

Hybrid hydrogels integrate natural and synthetic polymers or combine organic polymers with inorganic materials to synergistically leverage the advantageous properties of each component. This approach enhances hydrogel performance by simultaneously optimizing biocompatibility, mechanical strength, bioactivity, and biodegradability [15,70]. For example, hydrogels combining natural polymers such as gelatin or hyaluronic acid with synthetic polymers like PEG or PVA exhibit improved mechanical properties, controllable degradation rates, and enhanced cellular interactions. These composites effectively overcome the individual limitations of each polymer class, broadening their clinical applications in complex tissue regeneration and sustained drug delivery [95]. Nanocomposite hydrogels represent a specialized subset of hybrid hydrogels, incorporating nanoparticles, nanofibers, or nanoclays into hydrogel matrices. Integrating nanoparticles (e.g., mesoporous silica, magnetic nanoparticles, graphene oxide) substantially improves mechanical properties, enables stimulus-responsive drug delivery, and creates multifunctional therapeutic platforms with applications in targeted chemotherapy, regenerative medicine, and biosensing [96,97]. Collectively, hybrid hydrogels address critical biomedical challenges by combining favorable physicochemical characteristics with biological functionality, providing versatile and tailored solutions for personalized medicine and advanced therapeutic applications [15].

### 3.4. Advanced Functional Hydrogel Designs

Recent progress in polymer science and biomaterial engineering has led to advanced hydrogel designs with enhanced functionalities. These developments aim to improve traditional hydrogel characteristics by providing precise control over structural integrity, responsiveness to biological stimuli, self-healing capabilities, and mechanical durability, thereby expanding their therapeutic potential and clinical applications [98].

#### 3.4.1. Stimulus-Responsive Hydrogels

Stimulus-responsive or “smart” hydrogels exhibit dynamic physicochemical changes upon exposure to specific biological or environmental triggers, such as pH, temperature, ionic strength, enzymatic activity, redox conditions, light, magnetic fields, or electrical stimuli. These responsive behaviors result primarily from specialized functional groups or cross-linking mechanisms strategically integrated within the polymer network [75]. For example, pH-responsive hydrogels incorporate ionizable groups (e.g., carboxylic acids, amines) to enable selective swelling or deswelling in environments with distinct pH values, making them particularly suitable for targeted drug delivery to acidic tumor sites or inflamed tissues. Thermo-responsive hydrogels, notably poly(N-isopropylacrylamide) (PNIPAAm)-based systems, undergo reversible sol–gel transitions around physiological temperatures, facilitating injectable formulations that solidify upon administration. Enzyme-responsive hydrogels include peptide sequences cleavable by proteases overexpressed in specific disease states, allowing precise, site-specific therapeutic release [99,100,101]. Collectively, incorporating stimulus-responsive features into hydrogels significantly enhances therapeutic precision, reduces off-target side effects, and supports highly tailored treatments, particularly beneficial in cancer therapy, wound healing, inflammation control, and localized pain management (Table 3) [102,103,104].

#### 3.4.2. Self-Healing Hydrogels

Self-healing hydrogels possess the intrinsic ability to autonomously repair structural damage following physical disruption, significantly extending their functional lifespan and reliability in dynamic biological environments. The underlying mechanism typically involves reversible non-covalent interactions, such as hydrogen bonds, ionic bonds, hydrophobic interactions, host-guest interactions, or dynamic covalent chemistries, such as Schiff base linkages and Diels-Alder reactions [104,105]. Recent studies have demonstrated that self-healing hydrogels can rapidly restore their mechanical properties and original structure after experiencing deformation or damage. These unique features make self-healing hydrogels particularly valuable in applications involving repeated mechanical stress, such as injectable drug delivery systems, soft robotics, biosensors, and dynamically loaded tissue engineering scaffolds, including cartilage and cardiac tissue regeneration [62,105]. The integration of self-healing capabilities substantially improves clinical outcomes by minimizing implant replacement frequency, enhancing patient compliance, and reducing the risks associated with repeated interventions.

#### 3.4.3. Mechanically Robust Hydrogels

Conventional hydrogels often exhibit limited mechanical strength and stability under physiological conditions, restricting their use in load-bearing biomedical applications. To overcome this, mechanically robust hydrogels have been developed using innovative designs such as double-network hydrogels, interpenetrating polymer networks (IPNs), and nanocomposite formulations [106,107]. Double-network hydrogels typically combine rigid polymer networks with flexible polymer chains, resulting in enhanced toughness, strength, and fatigue resistance. Similarly, IPNs involve interweaving two or more independently cross-linked polymer networks, synergistically improving their mechanical resilience and durability [107,108]. These mechanically enhanced hydrogels significantly broaden clinical applications, including cartilage and bone regeneration, artificial ligaments, and durable wound dressings. Current research continues to refine mechanical properties while maintaining essential biological compatibility and functionality [109,110,111].

#### 3.4.4. Hybrid and Nanocomposite Hydrogels

Hybrid and nanocomposite hydrogels combine natural and synthetic polymers or incorporate nanoscale materials such as nanoparticles, nanofibers, or nanoclays into polymer matrices. These advanced composite systems synergistically enhance biocompatibility, mechanical strength, biodegradation control, and stimulus-responsive drug release profiles [70]. Incorporating inorganic nanoparticles such as silica nanoparticles, hydroxyapatite, graphene oxide, magnetic nanoparticles, and metallic nanoparticles (gold, silver) imparts multifunctionality, including targeted drug delivery, improved mechanical strength, enhanced conductivity, and controlled responsiveness. For instance, hydrogels embedded with magnetic nanoparticles enable externally controlled drug release and precise spatial targeting using magnetic fields, advantageous for targeted therapy and minimally invasive procedures [112,113]. Hybrid hydrogels that combine synthetic polymers (e.g., polyethylene glycol or polyacrylamide) with natural polymers (e.g., gelatin, hyaluronic acid) significantly improve biological interactions and mechanical properties. This approach addresses inherent limitations of each polymer type, such as the limited bioactivity of synthetic polymers or the mechanical weakness of natural polymers [15,70,114]. Nanocomposite hydrogels utilizing clay nanoparticles or nanofibers provide notable mechanical reinforcement, enhanced stimulus responsiveness, and improved drug delivery characteristics. These materials exhibit significant potential for diverse biomedical applications, including wound healing, sustained therapeutic delivery, tissue regeneration, biosensing, and precision medicine [15,70].

### 3.5. Drug Encapsulation and Release Mechanisms in Hydrogels

**Figure 3:**
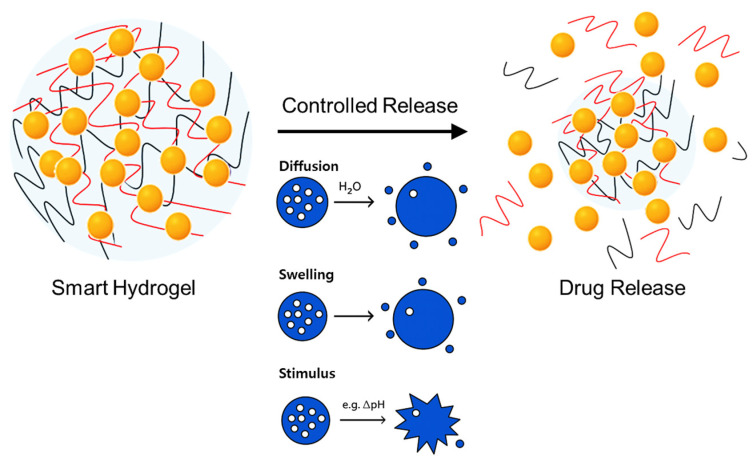
Schematic illustration of drug release mechanism in smart hydrogel.

Hydrogels are advanced carriers for therapeutic agents due to their excellent ability to encapsulate, protect, and sustainably release diverse bioactive molecules. Effective hydrogel-based drug delivery fundamentally relies on two critical processes: drug encapsulation and subsequent controlled release. A detailed understanding of these mechanisms is essential to optimize therapeutic efficacy and tailor hydrogels for specific clinical applications (Figure 3) [10,15,49,81,115].

#### 3.5.1. Encapsulation Principles

Drug encapsulation in hydrogels involves efficient loading and stable retention of therapeutic agents within the polymeric matrix. The encapsulation process depends primarily on polymer properties, cross-linking methods, drug characteristics (e.g., molecular weight, hydrophilicity/hydrophobicity, charge), and chosen encapsulation techniques. Common strategies include physical entrapment, chemical conjugation, and nanoparticle-mediated encapsulation. Physical entrapment involves incorporating drugs into hydrogel matrices during cross-linking or swelling, relying mainly on diffusion and non-covalent interactions. Although straightforward and preserving drug bioactivity, this method often exhibits limited control over initial burst release kinetics [116,117]. Conversely, chemical conjugation covalently attaches therapeutic molecules to hydrogels via cleavable or stimulus-sensitive linkages, enabling controlled and responsive release under specific physiological triggers (e.g., enzymes, pH changes, redox conditions) [118,119]. Nanoparticle-mediated encapsulation employs polymeric nanoparticles, liposomes, micelles, or inorganic nanoparticles integrated within hydrogels, significantly enhancing loading efficiency, stability, and targeted localization, especially for hydrophobic drugs or sensitive biomolecules. Nanoparticle-hydrogel composites are particularly advantageous in targeted chemotherapy, sustained release formulations, and personalized therapeutic strategies [120]. Overall, selecting an appropriate encapsulation method depends on therapeutic application, drug attributes, hydrogel characteristics, and desired drug-release profiles.

#### 3.5.2. Drug Release Mechanisms

Controlled drug release from hydrogels is critical for their effectiveness as drug delivery systems. Release kinetics primarily depend on physicochemical properties of the hydrogel and encapsulated drugs, polymer network structure (porosity, cross-link density, hydrophilicity), encapsulation methods, and environmental stimuli [121,122]. Diffusion-controlled release is the simplest and most common mechanism, driven by drug concentration gradients between hydrogel matrices and surrounding environments. Drug diffusion occurs through water-filled pores (Fickian diffusion) or via polymer relaxation and swelling dynamics (non-Fickian/anomalous diffusion). Adjusting porosity, cross-link density, and polymer composition enables precise control over diffusion rates, facilitating predictable and sustained drug release [73,123]. Swelling-controlled release involves hydrogel expansion upon water absorption, triggering polymer relaxation and drug diffusion. This release mode closely correlates with hydrogel swelling behavior, offering sustained and controlled therapeutic delivery superior to simple diffusion. Swelling-controlled mechanisms are particularly useful for stimulus-responsive hydrogels activated by specific biological signals, such as pH changes at tumor sites [124,125]. Chemically controlled release relies on hydrogel degradation or cleavage of covalent bonds linking drugs within the matrix. Degradation can occur through hydrolytic, enzymatic, or oxidative pathways, enabling precise and responsive drug delivery triggered by physiological conditions. Enzyme-sensitive peptide linkers or hydrolytically labile ester bonds facilitate targeted and controlled therapeutic release [126,127]. Stimulus-responsive drug release mechanisms involve external triggers such as temperature, pH, ionic strength, enzymes, redox conditions, magnetic or electrical fields, and light. Thermo-responsive hydrogels exploiting LCST behavior release drugs upon temperature-induced sol–gel transitions near physiological conditions, ideal for injectable applications. Similarly, hydrogels embedded with magnetic nanoparticles enable remote drug release through external magnetic fields, providing precise spatiotemporal control [128,129]. Integrating multiple release mechanisms enables the development of sophisticated hydrogel systems tailored for precise, targeted, and personalized therapeutic delivery. For non-opioid analgesics, this ability to precisely tune drug diffusion and degradation rates is particularly critical. For example, PNIPAAm-based thermosensitive hydrogels enable injectable delivery of gabapentinoids with temperature-triggered gelation, ensuring localized and sustained analgesia, while enzyme-responsive systems allow site-specific release of NSAIDs or ketamine in inflamed tissues. Ongoing research continues to explore hybrid mechanisms and combined stimulus-responsive strategies, aiming to maximize clinical efficacy, minimize side effects, and enhance patient outcomes (Table 4).

### 3.6. Clinical Implications of Hydrogel-Based Sustained Release for Non-Opioid Analgesics

Hydrogel-based sustained release systems for non-opioid analgesics offer an innovative therapeutic strategy with significant clinical implications, providing multiple advantages over traditional drug delivery approaches. By overcoming pharmacokinetic limitations such as short biological half-lives, rapid systemic clearance, and inadequate localization at target sites, hydrogels enhance therapeutic efficacy, patient adherence, and clinical outcomes. These advancements are particularly valuable in addressing challenges posed by the ongoing global opioid crisis [48,130,131].

#### 3.6.1. Clinical Benefits of Hydrogel-Based Delivery Systems

A primary clinical advantage of hydrogel-based sustained release formulations is their ability to deliver consistent therapeutic drug concentrations directly at target sites, enhancing analgesic efficacy while minimizing systemic exposure and associated adverse effects. Such localized delivery notably reduces drug-related toxicities, including gastrointestinal, cardiovascular, and central nervous system side effects commonly linked to systemic administration of analgesics like NSAIDs, gabapentinoids, and antidepressants [92,132,133]. Furthermore, sustained release significantly decreases dosing frequency, thereby improving patient adherence, convenience, and comfort particularly critical for chronic pain management, postoperative analgesia, and persistent neuropathic or musculoskeletal conditions, where maintaining stable therapeutic drug levels and patient compliance are essential for optimal outcomes [134,135,136]. Hydrogel systems also support minimally invasive delivery methods, including injectable in situ-forming gels, topical formulations, and transdermal patches. These delivery modes substantially enhance patient comfort, reduce procedural complications, and facilitate outpatient or ambulatory pain management. For instance, injectable hydrogels enable precise spatial control of drug distribution, ensuring high local bioavailability and minimizing invasiveness associated with surgical implantation or repeated injections [130,137].

#### 3.6.2. Clinical Examples and Recent Advances

Recent clinical and preclinical studies have highlighted the therapeutic potential of hydrogel-based non-opioid analgesic delivery systems. NSAID-loaded hydrogels containing diclofenac or ibuprofen demonstrated prolonged analgesic effects and enhanced inflammation control in preclinical osteoarthritis, rheumatoid arthritis, and postoperative pain models compared to conventional oral or topical treatments. Clinically, diclofenac-loaded hydrogels effectively reduced joint swelling and pain severity in osteoarthritis patients, significantly improving quality of life while minimizing gastrointestinal side effects [138,139,140]. Gabapentinoid-loaded hydrogels, such as gabapentin and pregabalin formulations, also represent promising advances, particularly for neuropathic pain management. Localized administration of these hydrogels substantially reduced neuropathic pain intensity and frequency, significantly decreasing systemic side effects, including dizziness, sedation, and cognitive impairment. Recent perioperative studies investigating gabapentinoid hydrogels demonstrated their potential to mitigate chronic postoperative pain development, reinforcing their clinical value [17,32,141]. Similarly, antidepressant-loaded hydrogels containing agents such as amitriptyline or duloxetine have been evaluated for chronic neuropathic pain conditions. These systems provided sustained therapeutic efficacy, improved tolerability, and significantly reduced systemic exposure compared to traditional oral dosing, notably decreasing side effects such as sedation, dry mouth, and cardiovascular disturbances, thus enhancing patient compliance [141]. Ketamine-loaded hydrogel systems have increasingly gained clinical interest for managing chronic, refractory neuropathic pain and opioid-resistant conditions. Studies demonstrated notable opioid-sparing effects, improved pain outcomes, and reduced adverse reactions through targeted, localized ketamine delivery. Injectable ketamine-hydrogel formulations show particular promise in minimally invasive, targeted treatment of severe neuropathic pain conditions, including complex regional pain syndrome (CRPS) and chronic postoperative pain. Such precise local delivery enhances therapeutic efficacy, allows lower doses, and significantly mitigates systemic adverse events like cognitive impairment, psychotomimetic effects, sedation, and cardiovascular disturbances associated with systemic ketamine administration [142,143,144].

### 3.7. Hydrogel-Based Delivery of Non-Opioid Analgesics

#### 3.7.1. NSAID-Loaded Hydrogels

NSAID-loaded hydrogels have gained significant attention for enhancing pain relief and reducing inflammation through sustained and localized drug delivery. Studies involving common NSAIDs, such as diclofenac, ibuprofen, and naproxen, consistently demonstrate advantages over conventional oral or topical formulations. Diclofenac-loaded hydrogels, for example, exhibited prolonged analgesic effects, substantial reduction in joint swelling, and decreased inflammation in preclinical arthritis models. Similarly, ibuprofen-loaded hydrogels provided sustained analgesia and anti-inflammatory effects in postoperative and musculoskeletal pain models, improving patient comfort, reducing dosing frequency, and minimizing gastrointestinal and systemic adverse events compared to traditional methods [133,138,145]. Recent advancements have introduced stimulus-responsive NSAID-hydrogel systems, allowing more precise, targeted drug delivery. These responsive hydrogels optimize release kinetics, sustain therapeutic concentrations at target sites, and minimize off-target systemic exposure. Collectively, NSAID-loaded hydrogels represent a promising strategy for improving chronic and acute inflammatory pain management, enhancing therapeutic outcomes and patient adherence [145,146].

#### 3.7.2. Gabapentinoid-Loaded Hydrogels

Gabapentinoids, including gabapentin and pregabalin, encapsulated within hydrogels have shown considerable promise in managing neuropathic pain through sustained and controlled release. Recent preclinical studies demonstrated that gabapentinoid-loaded hydrogels maintain prolonged and stable therapeutic drug concentrations at targeted pain sites, significantly enhancing analgesic efficacy compared to conventional methods. These hydrogel systems improve bioavailability and minimize fluctuations in plasma drug levels, notably reducing systemic side effects commonly associated with oral gabapentinoids, such as dizziness, sedation, and cognitive impairment [141,147]. Advances in injectable and stimulus-responsive hydrogels further enhance the clinical applicability of gabapentinoids by enabling precise local delivery and dynamic release in response to pathological signals, such as inflammation or local pH variations. Thus, gabapentinoid-loaded hydrogels represent a promising therapeutic advancement, potentially transforming clinical neuropathic pain management by improving efficacy, patient adherence, and overall quality of life [148,149].

#### 3.7.3. Antidepressant-Loaded Hydrogels

Hydrogel systems loaded with antidepressants such as amitriptyline and duloxetine represent a promising approach for managing neuropathic and chronic pain conditions. Recent studies have demonstrated that these hydrogels sustain therapeutic drug concentrations directly at affected sites, significantly prolonging analgesic effects compared to conventional oral administration. In animal models of neuropathic pain, antidepressant-loaded hydrogels showed enhanced analgesic outcomes, substantially reduced systemic exposure, and fewer adverse effects commonly associated with oral antidepressants, including sedation, dizziness, dry mouth, and cardiovascular disturbances [37,48]. Furthermore, advances in stimulus-responsive hydrogels have enabled precise, on-demand drug release triggered by pathological signals, such as inflammatory enzyme activity or localized pH changes. Such targeted responsiveness ensures optimal therapeutic concentrations specifically at pain sites, maximizing efficacy and minimizing systemic side effects. Consequently, antidepressant-loaded hydrogels hold significant promise to enhance patient adherence, therapeutic effectiveness, and overall outcomes in chronic neuropathic pain management [38,150,151].

#### 3.7.4. Ketamine-Loaded Hydrogels

Ketamine-loaded hydrogels represent an innovative approach for pain management, especially effective in treating chronic neuropathic pain and conditions resistant to traditional analgesics, including opioids. Recent studies have shown that hydrogel-based ketamine delivery systems provide sustained, localized analgesia by maintaining consistent therapeutic concentrations directly at the administration site. This targeted delivery significantly enhances analgesic efficacy while markedly reducing systemic adverse effects typically associated with conventional ketamine use, such as sedation, cognitive impairment, and psychotomimetic symptoms [141,142]. Injectable and stimulus-responsive hydrogel formulations further enhance clinical utility by allowing precise control over drug release in response to environmental or pathological stimuli at pain sites. These advanced hydrogel systems offer notable benefits, including improved patient outcomes, reduced dosing frequency, decreased systemic exposure, and fewer adverse events, representing a highly promising therapeutic strategy for chronic and refractory pain management [149,152].

#### 3.7.5. Topical Analgesic-Loaded Hydrogels

Topical analgesics such as capsaicin and lidocaine have been effectively incorporated into hydrogel formulations, delivering localized analgesia. Capsaicin-loaded hydrogels reduce pain transmission in chronic neuropathic conditions, including postherpetic neuralgia and diabetic neuropathy, through sustained depletion of substance P from peripheral sensory neurons. Lidocaine-loaded hydrogels achieve local analgesia by blocking voltage-gated sodium channels, providing effective relief in localized neuropathic and musculoskeletal pain conditions [153,154,155]. Clinical and preclinical studies consistently highlight that hydrogel-based topical formulations offer superior analgesic effectiveness, prolonged pain relief, improved patient compliance, and significantly reduced systemic absorption compared to traditional topical preparations. Recent advancements, particularly stimulus-responsive hydrogel systems, further enhance therapeutic precision by enabling controlled, targeted drug release at pain sites, maximizing analgesic efficacy while minimizing systemic side effects [156].

#### 3.7.6. Cannabinoid-Loaded Hydrogels

Cannabinoid-loaded hydrogels have recently gained considerable attention for their therapeutic potential in chronic pain and inflammation management. Studies involving hydrogels encapsulating cannabinoids such as cannabidiol (CBD) and tetrahydrocannabinol (THC) have demonstrated sustained analgesic effects with significantly reduced systemic psychoactive side effects. Preclinical research highlights that cannabinoid-loaded hydrogels effectively maintain therapeutic drug concentrations at localized sites, significantly improving analgesic efficacy in chronic neuropathic and inflammatory pain compared to conventional administration methods [157,158]. Moreover, stimulus-responsive cannabinoid hydrogel systems have enhanced therapeutic precision, providing targeted drug release activated by specific pathological or physiological stimuli. These advanced hydrogel formulations hold great promise as therapeutic strategies, effectively addressing existing limitations in chronic pain and inflammation management, while improving patient safety, adherence, and overall treatment outcomes (Table 5) [159].

In preclinical arthritis models, diclofenac- or CBD-loaded hydrogels demonstrated up to 60% reduction in inflammatory swelling and significant decreases in pain-related behaviors compared to conventional drug formulations. Similarly, gabapentin-loaded hydrogels maintained analgesic efficacy for over 72 h, whereas topical gels showed less than 12 h duration, underscoring the therapeutic advantage of hydrogel-based sustained delivery.

## 4. Challenges and Innovations in Hydrogel Non-Opioid Analgesics Systems

### 4.1. Optimizing Drug Loading and Sustained Release

Efficient drug loading and precise control over sustained drug release from hydrogel systems remain significant scientific and technical challenges. Achieving optimal drug loading efficiency requires careful consideration of hydrogel composition, polymer selection, encapsulation techniques, and cross-linking density. Recent research has focused on advanced strategies to enhance drug loading, including chemical conjugation of therapeutic agents to hydrogel networks, microencapsulation techniques to improve drug stability, and incorporation of nanoparticles to increase loading capacity and modulate release profiles [160,161]. Additionally, stimulus-responsive hydrogels have emerged as promising platforms for achieving precise and tailored drug release. These intelligent hydrogels selectively release therapeutics in response to specific physiological triggers, such as pH variations, temperature fluctuations, enzymatic activity, ionic strength changes, or pathological biochemical signals. Such controlled release strategies optimize therapeutic outcomes by maintaining sustained and localized drug concentrations, maximizing therapeutic efficacy, and minimizing systemic side effects. Continued advancements in hydrogel fabrication and characterization methods further support the development of sophisticated drug delivery systems, enabling personalized therapeutic approaches for chronic and acute pain management (Table 6) [146,162].

Compared with lipid nanoparticles and microneedle-based systems, hydrogel-based delivery platforms offer superior biocompatibility, prolonged residence time, and higher loading capacity for hydrophilic analgesic drugs. Moreover, their adjustable cross-linking density allows for tunable and predictable release kinetics, which is difficult to achieve in lipid or microneedle systems. These advantages collectively support the unique suitability of hydrogels for sustained and localized analgesic delivery.

### 4.2. Biocompatibility and Safety Concerns

Biocompatibility is essential for the successful clinical translation of hydrogel-based drug delivery systems. Despite promising initial outcomes, concerns persist regarding immunogenic reactions, cytotoxicity, and inflammation caused by hydrogel degradation products or residual cross-linking agents. Comprehensive biocompatibility evaluations including cytotoxicity testing, immunological assessments, and long-term in vivo safety studies are crucial to establish robust safety profiles. Additionally, systematic clinical trials are required to fully elucidate the long-term biological interactions and safety implications of hydrogels [62,163]. Innovations in hydrogel design increasingly incorporate naturally derived polymers and bioactive materials, such as hyaluronic acid, gelatin, and collagen derivatives, to enhance intrinsic biocompatibility and minimize adverse reactions. Advanced modifications, including surface functionalization and integration of anti-inflammatory agents, are actively pursued to further reduce immune responses and improve seamless integration within host tissues. Addressing these safety considerations through rigorous scientific assessment and strategic material development is critical for facilitating smooth clinical translation and accelerating the adoption of hydrogel-based therapies in effective and safe pain management [164].

### 4.3. Mechanical Stability in Dynamic Environments

Hydrogel applications in dynamic physiological environments such as joints, musculoskeletal tissues, and cardiovascular systems require superior mechanical stability, elasticity, and durability. Traditional hydrogels often exhibit mechanical fragility, rapid degradation, and insufficient resilience under physiological stress. To overcome these limitations, recent research has extensively explored hybrid or composite hydrogels reinforced with nanomaterials such as carbon nanotubes, graphene oxide, nanoclays, or polymeric nanoparticles. These composite strategies significantly enhance mechanical properties, including strength, elasticity, and fatigue resistance, while preserving essential therapeutic functions [165,166]. Additionally, innovations such as double-network hydrogels, interpenetrating polymer networks (IPNs), and covalent adaptable networks (CANs) have been developed, demonstrating remarkable mechanical robustness and self-healing capabilities. Such advanced hydrogel constructs effectively maintain structural integrity within dynamic biological conditions, broadening their clinical applicability in demanding therapeutic scenarios. Ongoing research continues refining composite and hybrid hydrogel designs to optimally balance mechanical performance, therapeutic efficacy, and biocompatibility, thereby enhancing clinical feasibility and therapeutic outcomes [167,168].

### 4.4. 3D-Printed and Customized Hydrogel Systems

Recent developments in additive manufacturing and 3D-printing technologies have markedly advanced hydrogel-based drug delivery, enabling personalized and patient-specific therapeutic solutions. Customizable 3D-printed hydrogels allow precise control over physical features, mechanical properties, drug release kinetics, and anatomical conformity tailored to individual patient needs, thereby significantly enhancing therapeutic efficacy and patient compliance [169,170]. Innovations in bioink formulations, enhanced printing resolutions, advancements in bioprinter technologies, and sophisticated computational modeling have contributed to developing refined hydrogel constructs. These technological improvements facilitate the fabrication of complex hydrogel architectures, ensuring precise drug localization, sustained release profiles, and responsiveness to physiological conditions. As 3D-printing technologies continue to evolve, personalized hydrogel systems hold substantial promise for clinical implementation in personalized medicine, potentially revolutionizing pain management and other therapeutic applications through customized, patient-centric treatments [171,172].

### 4.5. Translational and Manufacturing Challenges

The successful clinical translation of hydrogel-based drug delivery systems involves overcoming multiple translational and manufacturing barriers. Key challenges include ensuring reproducibility, scalability, batch-to-batch consistency, and compliance with stringent regulatory requirements. Variability in raw material quality, processing parameters, and environmental conditions complicates consistent manufacturing outcomes. Addressing these issues requires rigorous standardization protocols, advanced manufacturing methods, robust quality assurance systems, and comprehensive documentation [173,174]. Effective collaboration among academia, industry partners, and regulatory bodies is crucial to streamline approval processes and overcome translational hurdles. Adopting Good Manufacturing Practices (GMP), robust process validation approaches, and real-time monitoring technologies are essential strategies for improving reproducibility and scalability. Additionally, advancements in automated and continuous manufacturing processes offer promising avenues to enhance production efficiency and product consistency, facilitating broader clinical implementation of hydrogel-based therapeutics [175,176].

#### Regulatory Landscape of FDA-Approved Hydrogel Analgesic Systems

Several hydrogel-based analgesic systems have received FDA approval, highlighting their clinical feasibility. For example, Lidoderm^®^ (lidocaine patch) and Exparel^®^ (liposomal bupivacaine) employ hydrogel or hydrogel-like matrices for prolonged local analgesia. These approvals demonstrate the regulatory acceptance of hydrogel-based formulations, provided that biocompatibility, degradation rate, and controlled-release profiles are well characterized. Understanding such regulatory precedents is critical for designing next-generation non-opioid hydrogel systems.

### 4.6. Sustainability and Ethical Considerations

Sustainability and ethical considerations are critical in the development and clinical translation of hydrogel-based therapeutic systems. Employing environmentally sustainable and biodegradable materials, such as naturally derived polymers and renewable resources, significantly reduces ecological impact and aligns research with global sustainability objectives. Equally important are ethical research practices, transparency in product development, and strict adherence to patient-informed consent guidelines, ensuring responsible progression of hydrogel technologies [177,178]. Furthermore, promoting equitable access to advanced hydrogel therapeutics highlights the need for fairness and inclusivity in healthcare innovation. Continuous collaboration among researchers, clinicians, policymakers, and community stakeholders is essential to address potential disparities in healthcare accessibility. Actively advocating sustainability, ethical transparency, and equitable healthcare access enhances public trust, supports broader clinical adoption, and secures the long-term acceptance and success of hydrogel-based non-opioid analgesic therapies [179,180].

From a translational perspective, hydrogel-based analgesic systems are entering a promising phase of clinical evaluation. The global hydrogel drug delivery market is projected to expand significantly, driven by advances in polymer chemistry and minimally invasive administration routes. However, large-scale manufacturing, stability control, and regulatory harmonization remain key challenges. Addressing these issues through interdisciplinary collaboration will accelerate the market readiness of hydrogel-based non-opioid analgesic therapies.

### 4.7. Interdisciplinary Collaboration and Future Directions

Progress in hydrogel-based non-opioid analgesic systems relies heavily on interdisciplinary collaboration among anesthesiologists, bioengineers, materials scientists, pharmacologists, regulatory experts, and clinicians. Integrating diverse expertise and promoting collaborative research accelerates innovation, addresses complex translational challenges, and facilitates streamlined clinical implementation. Effective interdisciplinary communication supports the development of advanced, clinically viable hydrogel systems specifically tailored to therapeutic needs [136]. Future research should focus on strengthening interdisciplinary partnerships, optimizing regulatory pathways, and employing advanced computational modeling for predictive hydrogel design. To provide a clearer comparison of existing delivery technologies, a summary table (Table 7) has been added below to highlight the relative advantages and limitations of hydrogels versus other drug delivery platforms. Innovative platforms, including stimulus-responsive and self-healing hydrogels, represent promising strategies for enhancing therapeutic efficacy and patient adherence. Furthermore, advanced biofabrication techniques such as 3D printing and personalized medicine approaches can significantly improve hydrogel performance and expand clinical applications. Together, these collaborative efforts and forward-looking strategies will help overcome current limitations and substantially advance hydrogel-based analgesic therapies, ultimately improving patient outcomes and transforming pain management practices (Table 8) [181,182].

## 5. Conclusions

Hydrogel-based delivery systems for non-opioid analgesics represent a transformative approach in pain management, offering significant improvements over conventional therapies. These advanced systems facilitate precise, localized, and sustained drug release, substantially enhancing therapeutic efficacy, reducing systemic adverse effects, and notably improving patient compliance and comfort. Despite considerable progress, critical challenges remain, including optimization of drug loading and release kinetics, ensuring long-term biocompatibility, achieving mechanical stability under dynamic physiological conditions, addressing scalability in manufacturing, and navigating ethical and regulatory complexities. Successfully overcoming these barriers will require extensive interdisciplinary collaboration, innovative materials science integration, advanced manufacturing technologies, and refined regulatory frameworks. Ongoing research and development of hydrogel-based analgesic systems hold substantial promise for revolutionizing pain management practices, ultimately enabling personalized, effective, and patient-centered therapeutic interventions in anesthesiology and beyond. In conclusion, hydrogel-based non-opioid analgesic platforms represent a paradigm shift in pain management, offering safer and more effective alternatives to conventional opioid therapies.

## Figures and Tables

**Table 1:** Classification and mechanisms of major non-opioid analgesic drugs, including representative agents, primary modes of action, clinical applications, and key limitations.

Drug Class	Representative Agents	Mechanism of Action	Clinical Applications	Limitations
NSAIDs	Ibuprofen, Diclofenac	COX inhibition → ↓ prostaglandin synthesis	Inflammation, osteoarthritis, postoperative pain	GI irritation, renal toxicity
Acetaminophen	Paracetamol	Central COX inhibition, serotonergic/endocannabinoid modulation	Mild–moderate pain, fever	Hepatotoxicity (high dose)
Anticonvulsants	Gabapentin, Pregabalin	Bind α2δ subunit of calcium channels	Neuropathic pain, fibromyalgia	Sedation, dizziness
Antidepressants	Amitriptyline, Duloxetine	Modulate serotonin & norepinephrine pathways	Neuropathic pain, CRPS	CV risks, dry mouth, sedation
Anticonvulsants	Carbamazepine, Lamotrigine	Na^+^ channel inhibition, GABA enhancement	Neuropathic pain, trigeminal neuralgia	Cognitive side effects
NMDA antagonists	Ketamine	Block NMDA receptor → ↓ central sensitization	Refractory pain, CRPS	Psychomimetic effects
Topical agents	Capsaicin, Lidocaine	Substance P depletion/Na^+^ channel block	Localized neuropathic pain	Local irritation
Cannabinoids	THC, CBD	CB1/CB2 receptor modulation	Neuropathic pain, MS spasticity	Cognitive/psychoactive effects

**Table 2:** Types and characteristics of hydrogels used in biomedical applications, highlighting natural, synthetic, and hybrid categories with their advantages and limitations.

Type	Examples	Advantages	Limitations
Natural	Alginate, Chitosan, Hyaluronic acid, Collagen	Biocompatible, ECM-like, degradable	Mechanical weakness, batch variability
Synthetic	PEG, PVA, PLGA, PNIPAAm	Tunable mechanics, reproducibility	Lack of bioactivity
Hybrid	Gelatin-PEG, Nanocomposites	Combines strengths of natural/synthetic	Complex synthesis

**Table 3:** Overview of stimulus-responsive hydrogels, illustrating their mechanisms of action, representative materials, and clinical applications.

Stimulus	Representative Materials	Mechanism	Clinical Application
pH-responsive	Poly(acrylic acid), Chitosan	Ionization-driven swelling	Tumor, inflammatory tissue
Thermo-responsive	PNIPAAm, Pluronic F127	LCST sol–gel transition	Injectable depots
Enzyme-responsive	MMP-cleavable hydrogels	Enzyme-triggered degradation	Cancer, wound healing
Light-responsive	Photocrosslinkable gels	Controlled release under irradiation	On-demand drug delivery
Redox-responsive	Disulfide-linked gels	Cleaved by GSH	Intracellular delivery
Magnetic/electric	Magnetic NP gels, Conductive polymers	External field control	Targeted therapy

**Table 4:** Encapsulation strategies and drug release mechanisms in hydrogel-based delivery systems, summarizing diffusion-controlled, swelling-controlled, chemically controlled, and stimulus-responsive modes.

Mechanism	Description	Example	Advantages	Limitations
Diffusion-controlled	Drug release via concentration gradient	NSAID hydrogels	Simple, predictable	Burst release
Swelling-controlled	Polymer expansion drives release	PNIPAAm gels	Sustained release	Depends on swelling rate
Chemically controlled	Covalent linkages degrade	Enzyme-responsive gels	Precise targeting	Complex synthesis
Stimulus-responsive	pH, temp, magnetic, etc.	Ketamine gels	On-demand release	Need external trigger

**Table 5:** Clinical and preclinical applications of hydrogel-based delivery systems for non-opioid analgesics, with examples of drugs, formulations, conditions treated, and therapeutic outcomes.

Drug	Formulation	Model/Condition	Outcome
Diclofenac	Hydrogel patch	Osteoarthritis (clinical)	Pain reduction, ↓ GI side effects
Gabapentin	Hydrogel gel	Neuropathic pain (preclinical)	Sustained analgesia, ↓ sedation
Amitriptyline	Injectable hydrogel	Neuropathic pain (preclinical)	Prolonged effect, ↓ systemic toxicity
Ketamine	Injectable hydrogel	CRPS, chronic pain	Localized relief, ↓ psychomimetic effects
Lidocaine	Hydrogel patch	Neuropathic pain	Effective local anesthesia
CBD	Alginate-copper hydrogel	Arthritis, bone defect	Reduced inflammation, sustained effect

**Table 6:** Key challenges and current strategies in hydrogel-based non-opioid analgesic delivery systems, including drug loading, biocompatibility, mechanical stability, manufacturing, and ethical considerations.

Challenge	Limitation	Current Strategy	Future Direction
Drug loading	Low encapsulation efficiency	Nanoparticle integration, chemical conjugation	Personalized optimization
Biocompatibility	Immune reaction, cytotoxicity	Natural polymers, biofunctionalization	Long-term safety trials
Mechanical stability	Fragile under stress	Double-network, IPN, nanocomposites	Smart adaptive hydrogels
Manufacturing	Reproducibility, scalability	GMP, continuous manufacturing	Automated 3D-printing
Ethics & access	Sustainability, fairness	Biodegradable materials	Equitable access policies

**Table 7:** Comparison of major drug delivery platforms for analgesia.

Delivery Platform	Mechanism/Carrier type	Release Characteristics	Advantages	Limitations
Hydrogels	3D polymer networks	Sustained, localized	Biocompatible, tunable, minimally invasive	Mechanical weakness
Lipid nanoparticles	Lipid core–shell carriers	Rapid or burst release	High permeability, suitable for lipophilic drugs	Stability and aggregation issues
Microneedles	Transdermal micro-protrusion	Pulsatile or rapid onset	Painless delivery, self-administration	Limited drug load, potential irritation
Polymeric depots	PLGA-based injectable systems	Long-term sustained release	Clinically approved (DepoFoam, etc.)	Limited for hydrophilic drugs

**Table 8:** Future directions and interdisciplinary strategies for advancing hydrogel-based non-opioid analgesic therapies, emphasizing innovations in 3D printing, self-healing hydrogels, nanocomposites, regulatory science, and collaboration.

Focus Area	Current Progress	Future Outlook
3D printing	Patient-specific hydrogel structures	Personalized medicine platforms
Self-healing gels	Injectable, stress-resistant hydrogels	Long-term implants, robotics
Nanocomposite hydrogels	Drug co-delivery + tissue regeneration	Theranostic platforms
Regulatory science	Preclinical trials, limited approvals	Streamlined FDA/EMA pathways
Interdisciplinary collaboration	Material scientists + clinicians	Translational consortia

## Data Availability

The datasets generated and/or analysed during the current study are not publicly available because disclosing patients’ personal information is against the law but only de-identified datasets are available from the corresponding author on reasonable request.
